# Hybrid Surgery for Severe Mitral Valve Calcification: Limitations and Caveats for an Open Transcatheter Approach

**DOI:** 10.3390/medicina58010093

**Published:** 2022-01-07

**Authors:** Erik Bagaev, Ahmad Ali, Shekhar Saha, Sebastian Sadoni, Martin Orban, Michael Naebauer, Julinda Mehilli, Steffen Massberg, Andreas Oberbach, Christian Hagl

**Affiliations:** 1 German Centre for Cardiovascular Research (DZHK), Department of Cardiac Surgery, Ludwig Maximilian University of Munich, Partner Site Munich Heart Alliance, 80539 Munich, Germany; erik.bagaev@klinikum-nuernberg.de (E.B.); ahmad.ali@med.uni-muenchne.de (A.A.); sebastian.sadoni@med.uni-muenchen.de (S.S.); andreas.oberbach@medizin.uni-leipzig.de (A.O.); christian.hagl@med.uni-muenchen.de (C.H.); 2 Department of Cardiac Surgery, Klinikum Nürnberg, Paracelsus Medical University, 90471 Nuremberg, Germany; 3 German Centre for Cardiovascular Research (DZHK), Department of Cardiology, Ludwig Maximilian University of Munich, Partner Site Munich Heart Alliance, 80539 Munich, Germany; martin.orban@med.uni-muenchen.de (M.O.); michael.nabauer@med.uni-muenchen.de (M.N.); julinda.mehilli@lakumed.de (J.M.); Steffen.massberg@med.uni-muenchen.de (S.M.); 4 Medizinische Klinik I, Landshut-Achdorf Hospital, 84036 Landshut, Germany

**Keywords:** transcatheter heart valves, mitral valve, mitral annular calcification

## Abstract

*Background and Objectives*: Mitral stenosis with extensive mitral annular calcification (MAC) remains surgically challenging in respect to clinical outcome. Prolonged surgery time with imminent ventricular rupture and systolic anterior motion can be considered as a complex of causal factors. The aim of our alternative hybrid approach was to reduce the risk of annual rupture and paravalvular leaks and to avoid obstruction of the outflow tract. A review of the current literature was also carried out. *Materials and Methods:* Six female patients (mean age 76 ± 9 years) with severe mitral valve stenosis and severely calcified annulus underwent an open implantation of an Edwards Sapien 3 prosthesis on cardiopulmonary bypass. Our hybrid approach involved resection of the anterior mitral leaflet, placement of anchor sutures and the deployment of a balloon expanded prosthesis under visual control. Concomitant procedures were carried out in three patients. *Results:* The mean duration of cross-clamping was 95 ± 31 min and cardiopulmonary bypass was 137 ± 60 min. The perioperative TEE showed in three patients an inconspicuous, heart valve-typical gradient on all implanted prostheses and a clinically irrelevant paravalvular leakage occurred in the anterior annulus. In the left ventricular outflow tract, mild to moderately elevated gradients were recorded. No adverse cerebrovascular events and pacemaker implantations were observed. All but one patient survived to discharge. Survival at one year was 83.3%. *Conclusions:* This “off label” implantation of the Edwards Sapien 3 prosthesis may be considered as a suitable bail-out approach for patients at high-risk for mitral valve surgery or deemed inoperable due to extensive MAC.

## 1. Introduction

Mitral annular calcification (MAC) with an estimated prevalence of 8–42%, is related to the degree of aortic, coronary artery, as well as aortic valve calcification, and has been associated with an increased atherosclerotic burden, adverse cerebrovascular events and cardiovascular mortality [1]. About 20% of patients undergoing mitral valve surgery due to mitral stenosis exhibit varying degrees of MAC [2].

The surgical treatment of severe MAC remains a technical challenge, especially from a surgical point of view [3]. The massive calcification of the posterior mitral valve annulus does not allow for conventional implantation of a mitral valve prosthesis. In these cases, calcification is intramural and focal decalcification is associated with weakening of the mitral annulus and a significant risk of rupture of the atrio-ventricular junction, dissection of the left ventricular wall and injury to the circumflex artery [2,4,5]. In order to preserve the mitral annulus and avoid complications, the role of transcatheter heart valves (THV) has been investigated in these patients [6,7,8,9]. The challenges of a THV essentially relate to the anchoring of the THV in the incompletely calcified annulus, with the subsequent increased risk of valve dislocation or paravalvular leakage [10]. Even in the case of ideal positioning of the mitral valve prosthesis, the additive risk of a systolic anterior movement (SAM), left ventricular out flow tract obstruction (LVOTO) due to high profile of the prothesis, prosthesis stability and wire entrapment in the chordae apparatus remains [5,10,11,12].

Patients presenting with MAC often exhibit only a partially calcified annulus. This offers insufficient anchorage for the balloon-expanded in the soft structural components of mitral valve anulus, which offers low stability for the THV. This clinical challenge with the increased risk of para-valvular leakage has not yet been adequately addressed. Previous reports on THVs in the treatment of patients with MAC have reported several complications, one of note being paravalvular leakage [5,6,10]. This necessitates the use of adjunctive procedures to avoid such complications [13]. A possible therapeutic approach could be the open implantation of an Edwards Sapien 3 prosthesis with simultaneous anchoring of the prosthesis ring as a bail-out approach in a hybrid surgical approach to reduce the risk of paravalvular leakage associated with the use of THVs in patients with MAC. Furthermore, as the current literature regarding the use of THV is sporadic, a review of the literature was carried out to identify reports detailing the use of THV in the setting of MAC.

## 2. Methods

### 2.1. Study Design

This study was approved by the Ethical Board of the Ludwig Maximilian University (No. 21-0327) and informed consent was waived. From December 2016 to February 2018, a total of six patients with high-grade mitral stenosis were treated with an open implantation of the Edwards Sapien 3 valve prosthesis (Edwards Lifesciences, Irvine, CA, USA). All patients had massive calcification of the posterior mitral valve annulus. Treatment strategy was discussed in advance for all patients in the interdisciplinary Heart Team. Postoperative treatment and data acquisition were performed as part of routine patient care. Data acquisition was based on our institutional database and then de-identified. Additionally, a review of the current literature was carried out.

### 2.2. Search Strategy

A systematic review of available data reporting outcomes on open transcatheter mitral valve implantation in the setting of severe mitral annular calcification was performed in accordance with the guidance and the reporting items specified on the Preferred Reported Items for Systematic Reviews and Meta-Analysis (PRISMA) statement [14]. A manual search was performed on databases in order to identify relevant entries. All publications were limited to those involving human subjects and in the English language. Emphasis was placed on the adjunctive measures and the valvular gradients and concomitant procedures.

### 2.3. Definition of Parameters

1. Diagnosis of valvular lesions was made in accordance with the ESC guidelines [15].
2. Adverse cerebrovascular events were defined as new-onset postoperative neurological symptoms, which were accompanied by a new computed tomography (CT) confirmed central nervous system (CNS) lesion [16].
3. Preoperative risk assessment was performed by determining the European System for Cardiac Operative Risk Evaluation II (EuroSCORE II).
4. Operative mortality was defined as in-hospital mortality and mortality within 30 days, regardless of cause.

### 2.4. Follow-Up

Follow-up was achieved by routine check-ups and patient interviews. Postoperative follow-up examinations were carried out at discharge after six months and then yearly. Echocardiographic data was collected at six-months and at one year.

### 2.5. Statistical Analysis

Data was analyzed using the IBM SPSS Statistics Data Editor^®^ version 25. Data are presented as mean ± standard deviation (SD) or as absolute numbers (percentages) unless otherwise specified.

## 3. Results

### 3.1. Patient and Imaging Characteristics

The mean age of the patient cohort was 76 ± 9 years with a mean EuroSCORE II of 5.7 ± 1.9 (Table 1). Severe aortic stenosis was diagnosed in two patients and coronary artery disease in one patient required myocardial revascularization (Table 1). Almost all patients had a high BMI and were obese. Chronic atrial fibrillation was seen in 4 patients. All patients suffered from multiple comorbidities such as renal insufficiency (*n* = 3) and lung function impairment (*n* = 6). No patient had had previous heart surgery.

**Figure 1:**
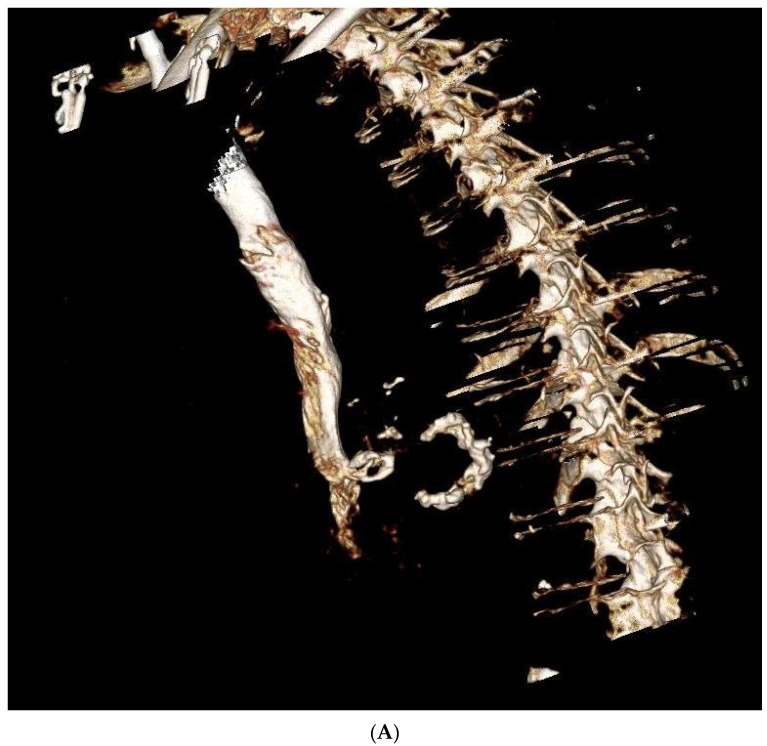

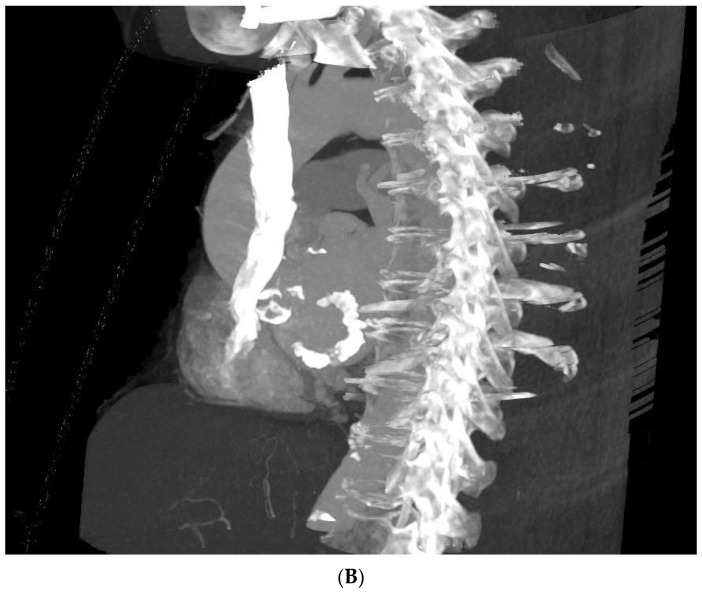
Computed Tomography showing extensive mitral annular calcification. (**A**) 3D recontruction of the calcified mitral annulus. (**B**) Computer tomography of the calcified mitral annulus.

The diagnosis of severe mitral stenosis was confirmed echocardiographically, and severe MAC was confirmed with computer tomography (CT). Additionally, severe aortic stenosis was diagnosed in two patients (Table 1). The average diastolic pressure gradient over the mitral valve was 26 ± 1.3 mmHg and the mean gradient was 11.7 ± 3.4 mmHg. The mean left ventricular pump function was 61.3 ± 11.6%. The calcification of the mitral valve annulus, as demonstrated by CT, was in all cases at least hemi-circular up to 2/3 of the circumference (Figure 1).

### 3.2. Operational Management

**Figure 2:**
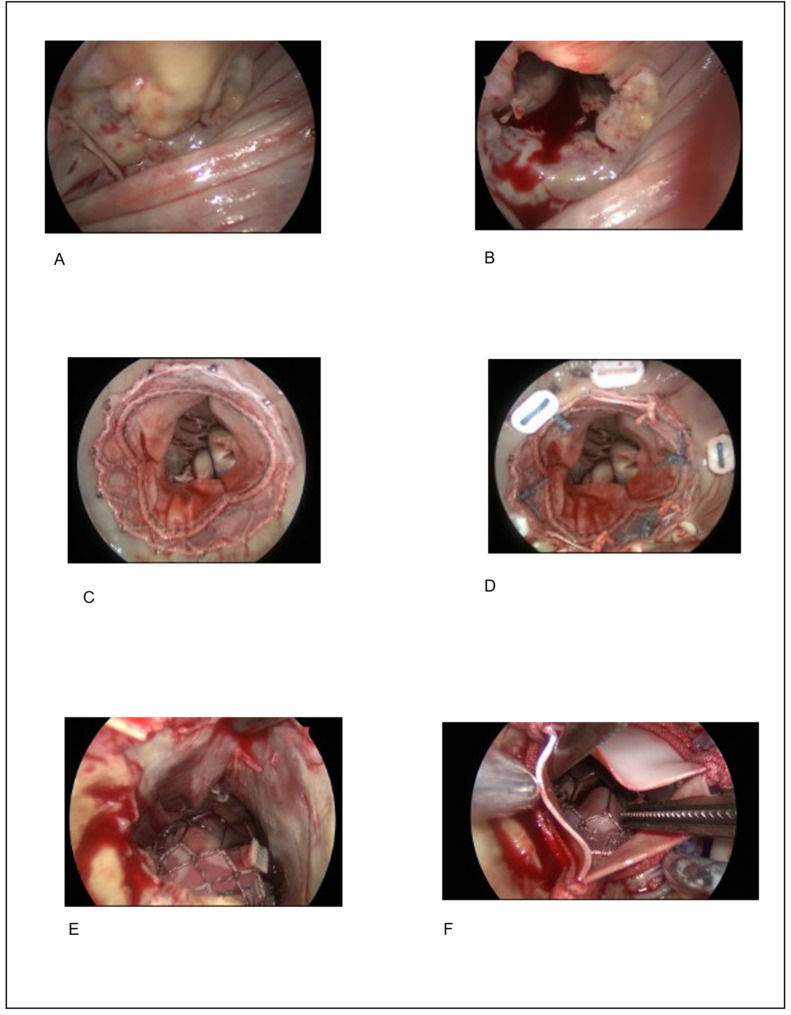
Stepwise placement of the prosthesis: (**A**) Mitral valve with calcified annulus. (**B**) Following resection of the AML. (**C**) Expansion of the valve without pledgets. (**D**) The prosthesis is anchored with pledget reinforced sutures. (**E**) View of the LVOT showing no signs of obstruction. (**F**) View of the LVOT following implantation of an aortic valve prosthesis showing no signs of obstruction. AML: Anterior mitral leaflet, LVOT: Left ventricular outflow tract.

The implantation was carried out in all patients via a median sternotomy on cardiopulmonary bypass. In all cases, access to the mitral valve was through the left-atrium (Figure 2A). Before the implantation of the Sapien 3 valve prosthesis in the mitral valve position, a partial or complete resection of the anterior mitral valve leaflet (Figure 2B) was generally performed to prevent systolic anterior motion of the anterior leaflet of the mitral valve. The mitral valve annulus was sized to estimate the size of the prosthesis using a measuring bell (Fehling Instruments, Karlstein am Main, Germany).

The primary challenge during THV implantation in the calcified annulus is to align the THV with the valve tissue and avoid dislocation of the prothesis in the non-calcified partition. To ensure this, anchoring sutures on the atrial side should be attached as close as possible to the THV to facilitate anchoring and adequate paravalvular sealing. The first patient received five anchoring sutures on the atrial side after prosthesis placement, approx. 1 cm before the calcified annulus. This allowed sufficient atrial tissue to be adapted to the prosthesis ring without compromising the atrial geometry. The following patient showed a good anatomical alignment of the calcified mitral annulus and the THV, so that initially no anchoring sutures seemed necessary (Figure 2C). Intraoperative echocardiography showed significant, para-valvular leaks in the anterior part of the annulus. Consequently, anchoring sutures had to be secondarily placed. The primary placement of anchoring sutures (Figure 2D) prior to implantation of the THV was performed in subsequent patients. Following this technique, no relevant paravalvular leakages were observed. The chordae of the posterior leaflet including the commissural junction remained intact.

**Figure 3:**
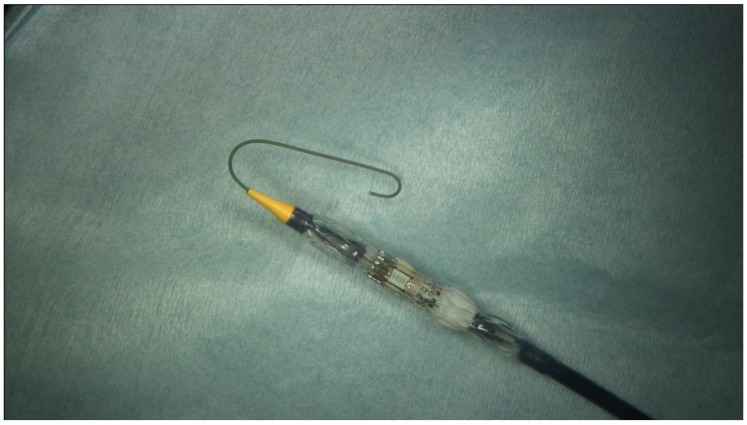
Modified delivery system.

The first implantation of the Sapien 3 prosthesis was accompanied by a perforation of the ventricle through the tip of the “delivery system” (Figure 3). The defect in the tip of the heart was then closed with several felt-reinforced sutures. To protect the ventricle, a soft wire was inserted into the delivery system (Figure 3) in the subsequent procedures to avoid the above-mentioned complication. Under view, the prosthesis in the mitral valve position could be expanded well using the modified “delivery system” (Figure 2D).

### 3.3. Concomitant Procedures

In two cases, a clinically relevant aortic valve stenosis also required an aortic valve replacement, which was carried out with a rapid deployment valve (Intuity Elite, Edwards Lifesciences) (Table 2). This allowed examination of the left ventricle outflow tract after implantation of the Sapien 3 prosthesis in the mitral valve position with regard to a possible dislocation or obstruction of the outflow tract through the prosthesis or AML tissue remnants. Both cases demonstrated sufficient patency of the outflow tract (Figure 2E,F). Concomitantly, in one case a Morrow myectomy was performed, while in another a double myocardial revascularization was performed, and in five cases an occlusion or amputation of the left atrial appendage was performed.

### 3.4. Postoperative Outcomes

The mean duration of aortic cross-clamping was 95.5 ± 30 min, with a mean duration of cardiopulmonary bypass of 137 ± 60 min. The perioperative transesophageal echocardiography showed valve-typical gradients (dPmax 11.5 ± 2.9 mmHg; dPmean 5.2 ± 1.3 mmHg) in all 6 cases. Postoperatively, only negligible paravalvular leaks was observed (Table 2). Echocardiographic diagnostics revealed a slight to moderate increase in the gradient above the outflow tract of LVOT (dPmax 25 ± 1.3 mmHg; dPmean 12.5 ± 2.9 mmHg). The average length of stay at the ICU was 9 ± 8 days and the average ventilation time was 76 ± 66 h (Table 2).

We observed no adverse cerebrovascular events. A total of two patients underwent surgical site re-exploration due to bleeding. Temporary renal-replacement therapy was required in three patients (50.0%). We observed no heart rhythm disturbances and none of the patients required a pacemaker postoperatively. All but one patient survived to discharge. The cause of mortality was multi-organ failure due to sepsis. The operative mortality was 16.7%.

### 3.5. Follow-Up

**Figure 4:**
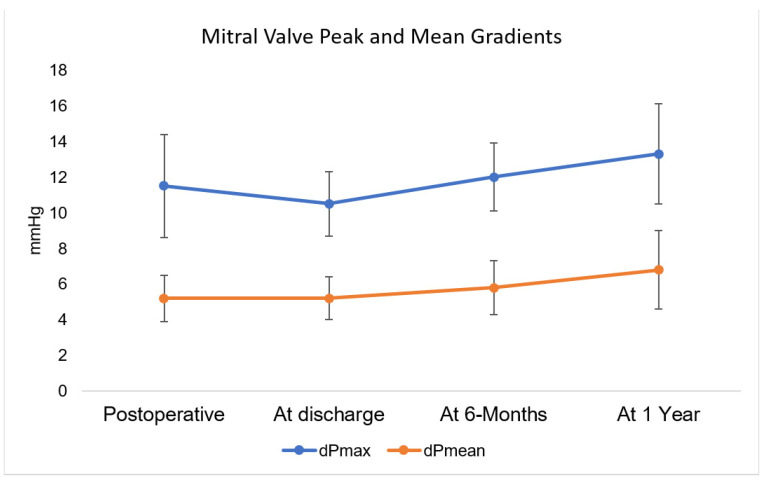
Mitral valve peak and mean gradients.

Echocardiographic data at discharge and follow-up is detailed in Figure 4. Mean mitral valve gradient was 6.8 ± 2.2 mmHg. All patients exhibited preserved left ventricular ejection fraction. Survival at one year was 83.3%. One patient was lost to follow-up at one year.

## 4. Discussion

Patients with mitral valve stenosis and accompanying advanced MAC are a complex treatment cohort. The therapy decision is additionally triggered by an accompanying multi-organ pathology, which increases both early postoperative and long-term mortality [1]. Traditional surgical management consists of an overly complex decalcification of the annulus with subsequent repair of the atrioventricular transition with a patch. A reconstruction of the anatomical defect caused by decalcification is only possible in long surgical interventions which increases the peri- and post-operative accompanying risk [17,18]. Progression of MAC is known to be associated with advanced age and renal failure and is recognized as a cardiovascular mortality predictor [19,20,21,22]. THVs have been described as viable alternatives to the classic surgical mitral valve replacement [23]. An optimal therapy option for such patients is one, which both guarantees a high level of patient safety with reduced surgical risk and at the same time takes into account the anatomical considerations defined by the pathology. The above described hybrid surgical approach is our modified approach to a direct transatrial implantation of balloon-expanding prothesis in the mitral valve position [8,9]. Furthermore, the additional adjunctive measeures have been listed in Table 3. 

### 4.1. Choice of Prosthesis

We preferred the Sapien 3 valve prosthesis (Edwards Lifesciences, Irvine, CA, USA) due to its lower crimping profile as compared to the Sapien XT prosthesis (6.7 mm v/s 8 mm) [43]. This allowed for a better positioning of the THV prosthesis. The additional outer polyethylene terephthalate cuff of the Sapien 3 provides an extended landing zone to enhance paravalvular sealing [44]. Since the frame is taller in case of the Sapien 3 in both crimped (20 mm v/s 17.2 mm) and expanded (28 mm v/s 20.1 mm) states, higher positioning of the THV prosthesis in the mitral position must be performed carefully to avoid LVOTO [44].

### 4.2. Operative Considerations

To this day, no standardized procedure has been established in clinical practice, since a large number of operational challenges have not yet been addressed, such as impending ventricular rupture and systolic anterior motion. Praz et al. and Russel et al. have described adjunctive procedures with the use of polytetrafluoroethylene (PTFE) strips to provide further stability to the annulus [6,13], whereas others report transcatheter mitral valve replacement without adjunctive procedures [5,12,23,41].

It is our opinion that the intervention should always be carried out on the cardiopulmonary bypass with adequate cardioplegia to enable the above-mentioned manipulation of the mitral valve and to reduce the risk of calculi or air embolism. In our cohort no adverse cerebrovascular events were observed. This offers the additional advantage to perform concomitant procedures as needed. To minimize the duration of cross clamping, rapid deployment valves in case of aortic valve stenosis should be considered.

The advantage of the hybrid surgical approach, in contrast to the completely catheter-based procedure, lies in the direct visualization of the prosthesis in the annulus, as well as in the accompanying resection of the anterior mitral valve leaflet to prevent SAM related obstruction of the left ventricular outflow tract [7]. An important advantage of using THVs in the setting of MAC is that there is no need of focal decalcification of the mitral valve annulus. Anterior mitral valve leaflets should be partially or completely resected to avoid the SAM phenomenon. Despite the above-mentioned improvements to the Sapien 3 prosthesis, it is our recommendation that the THV be additionally fastened with several anchoring sutures to reduce the frequency of the postoperative paravalvular leak. Furthermore, there have been reports of early and late migration of the THV implanted in the mitral position [35,42,45]. An added advantage of this hybrid approach is that it allows for fixation of the THV to the mitral annulus.

Prosthesis-patient mismatch (PPM) is an independent predictor of mortality after mitral valve replacement [46]. Optimal sizing is of utmost importance, especially in these cases, as oversizing the valve may lead to complications such as ventricular rupture and occlusion of the circumflex artery. An indexed valve effective orifice area (iEOA) of more than 1.2 cm^2^/m^2^ has been reported to be a not clinically significant PPM. The reference iEOA of the Sapien 3 (26 mm: 1.12 ± 0.19 cm^2^/m^2^, 29 mm: 1.28 ± 0.21 cm^2^/m^2^) deems it suitable for use in the mitral position without causing significant PPM [47].

Left ventricular outflow tract obstruction has been reported to occur in 13% and up to 22% among the patients undergoing transcatheter mitral valve replacement [48]. Measures used to prevent LVOTO include percutaneous laceration of the anterior mitral leaflet, pre-emptive alcohol, septal ablation and deployment of the THV within the AML [48,49]. However, these procedures include several risks, such as inability to prevent obstruction from the covered valve skirt, pacemaker implantation and uncontrolled ballooning in the AML. Therefore, a hybrid approach entailing surgical resection of the AML followed by deployment of the THV is our preferred method to avoid LVOTO. Positioning and deployment of the valve should be performed carefully, so that a larger portion of the Sapien prosthesis lies as far as possible on the atrial side during implantation. In cases where LVOTO is unavoidable, a hybrid approach allows for septal myectomy, as was the case in one of the patients in this cohort and has also been described by others [9,32].

Infective endocarditis has been reported to be the most common indication of surgery following aortic THV implantation [50]. Following THV implantation, vigilance, early diagnosis, adequate antibiotic treatment, and early surgery are essential [50,51] in cases of infective endocarditis.

### 4.3. Limitations

This study suffers from several limitations. The retrospective, single-centre design and the small number of patients are associated with a low power of the statistical analyses. Moreover, follow-up and primary end-points described here are short-term and mid-term. All patients in this cohort met at least one exclusion criteria as laid out by the Mitral Valve Academic Research Consortium (MVARC), so that the endpoints described by the MVARC were not considered [44]. Larger multi-center trials are required.

## 5. Conclusions

This hybrid implantation of a THV may be considered as a suitable bail-out approach for patients at high-risk for mitral valve surgery or deemed inoperable due to extensive MAC. The THVs exhibit acceptable gradients and the risk of LVOTO can be minimize through the hybrid procedure. Further technical advancements of the THVs with a lower profile and optimized delivery system would be advantageous for the treatment of this multimorbid cohort.

## Figures and Tables

**Table 1:** Demographic and Echocardiographic data.

Demographic Data	(*n* = 6)
Age (years)	76 ± 9
Female (%)	6 (100.0)
BMI (kg/m^2^)	29 ± 4.5
EuroSCORE II (%)	5.7 ± 1.9
NYHA III-IV (%)	6 (100.0)
Chronic kidney disease (%)	5 (83.3)
GFR (mL/min)	55 ± 22
Atrial fibrillation (%)	4 (66.7)
COPD (%)	3 (50.0)
FEV1 (l)	1.6 ± 0.2
Coronary artery disesase (%)	2 (33.3)
**Echocardiographic data**	
LVEF (%)	61.3 ± 11.6
Impaired RV function (%)	1 (16.7)
Severe aortic stenosis (%)	2 (33.3)
Mild to moderate aortic regurgitation (%)	1 (16.7)
Severe mitral regurgitation (%)	3 (50.0)
Severe mitral stenosis (%)	6 (100.0)
• MV dPmax (mmHg)	26 ± 1.3
• MV dPmean (mmHg)	11.7 ± 3.4
Moderate to severe Tricuspid regurgitation (%)	1 (16.7)

Data are presented as mean ± standard deviation (SD) or as absolute numbers (percentages). COPD: Chronic obstructive pulmonary disease, FEV1: forced expiratory volume in one minute, BMI: Body mass-index. GFR: Glomerular filtration rate, LVEF: Left ventricular ejection fraction, MV: Mitral valve, NYHA: New York Heart Association, RV: Right ventricle.

**Table 2:** Intraoperative and postoperative data.

Details of Surgery	(*n* = 6)
Duration of CPB (min)	137 ± 60
Cross-clamp time (min)	95 ± 31
Prosthesis size	
• 26 mm (%)	1 (16.7)
• 29 mm (%)	5 (83.3)
Concomitant procedures	
• Aortic valve replacement (%)	2 (33.3)
• Morrow resection (%)	1 (16.7)
• CABG (%)	1 (16.7)
• LAAO (%)	5 (83.3)
**Intraoperative Echocardiographic data**	
MV dP max (mmHg)	11.5 ± 2.9
MV dPmean (mmHg)	5.2 ± 1.3
LVOT dPmax (mmHg)	25 ± 1.3
LVOT dPmean (mmHg)	12.5 ± 2.9
None to trace paravalvular leakage	6 (100.0)
**Morbidities**	
Re-explorative surgery (%)	2 (33.3)
Surgical site infection (%)	1 (16.7)
Renal replacement therapy (%)	3 (50.0)
Nosocomial pneumonia (%)	2 (33.3)
**Outcomes**	
Duration of mechanical ventilation (hours)	76 ± 66
ICU stay (days)	9 ± 8
Total hospital stay (days)	26 ± 17
Operative mortality (%)	1 (16.7)

Data are presented as mean ± standard deviation (SD) or as absolute numbers (percentages). CABG: Coronary Artery Bypass Grafting, CPB: Cardiopulmonary Bypass, ICU: Intensive care unit, LAAO: Left atrial appendage occlusion, LVOT: Left ventricular outflow tract, MV: Mitral valve.

**Table 3:** Review of literature of hybrid approaches to transcatheter mitral valve replacement in the setting of mitral annular calcification. CABG: Coronary Artery Bypass Grafting, LAAE Left Atrial Appendage Excision, SAVR: Surgical Aortic Valve Replacement, TAVR: Transcatheter Aortic Valve Replacement, TVr: Tricuspid Valve repair.

Author	Year	Valve Implanted	Nr. of Patients	Access	Adjunctive Procedures	PVL	Postoperative Mitral DPmean (mmHg)	Concomitant Procedures
Carrel et al. [24] (Switzerland)	2012	Sapien XT	1	Median sternotomy On pump Transatrial access	Valve fixed with sutures to the annulus	No	2	-
Astarci et al. [25] (Belgium)	2013	Sapien XT	1	Median sternotomy On pump Transatrial access	Bovine pericardium was used to seal a paravalvular leakage	No	3	SAVR and CABG
Sinning et al. [26] (Germany)	2013	Sapien XT	1	Transapical access	None	Mild	3	-
Ferrari et al. [27] (Switzerland)	2013	Sapien XT	1	Right Thoracotomy On pump Transatrial access	Sutures at commissures	No	-	-
El-Eshmawi et al. [28] (USA)	2015	Melody valve	1	Median sternotomy On pump Transatrial access	None	No	3	TVr, LAAE, Ablation
Mellert et al. [29]	2015	Direct flow medical	1	Transapical	None	Mild	2	-
Lim et al. [30] (United Kingdom)	2015	Lotus	2	Transapical	None	Mild	Case 1: 4 Case 2: 7	-
Dahle et al. [9] (Norway)	2015	Sapien XT	1	Median sternotomy On pump Transatrial access	Anchoring sutures on the atrial wall	No	-	Modified Konno procedure with myectomy was performed later due to LVOT obstruction
Lee et al. [31] (USA)	2016	Sapien XT	1	Median sternotomy On pump Transatrial access	Anchoring sutures in the mitral annulus	Mild	2	SAVR
Langhammer et al. [32] (Switzerland)	2016	Sapien XT and Sapien 3	4	Transatrial and transseptal	In two cases additional suture fixation and in two cases use of xeno-pericardial patch to reduce paravalvular leakage	Mild (1)	Case 1: 5 Case 2: 5 Case 3: 4 Case 4: 4	Case 1: Maze procedure Case 3: CABG, septal myectomy
Koeckert et al. [33] (USA)	2016	Sapien XT	1	Robotic approach- On pump-Transatrial access	Placement of three periannular sutures	No	3	-
Baumgarten et al. [34] (USA)	2016	Sapien XT and Sapien 3	3	lateral mini- thoracotomy-on pump-Transatrial access	None	Mild (1)	Case 1: 2 Case 2: normal Case 3: 4	-
Murashita et al. [8] (USA)	2016	Sapien XT	1	Median sternotomy On pump Transatrial access	None	Trivial	2	-
Guerrero et al. [7] (Multicenter)	2016	Sapien XT, Sapien 3, Inovare	64	Direct open transatrial (9) Transapical (29) Transseptal (26)		mild or absent (95.1%), Severe (4.9%)	5.8 ± 2.2	TAVR (11) SAVR (6)
El Sabbagh et al. [5] (USA)	2017	Sapien XT and Sapien 3	6	Median sternotomy (3)/Right anterolateral thoracotomy (3)- On-pump- Transatrial (4)/Trans septal (2)	Anchoring stitches when necessary	Severe (3) Moderate (1)	5 ± 1	-
Ghosh-Dastidar et al. [35] (United Kingdom)	2017	Lotus	1	Initial transapical access followed by Median sternotomy- On pump- Transatrial access	Teflon collar and anchoring sutures	No	normal	-
Polomsky et al. [36] (USA)	2017	Sapien 3	2	Median sternotomy- On pump- Transatrial access	Sutures at commissures	No	-	SAVR (2)
Praz et al. [6] (USA)	2018	Sapien XT and Sapien 3	26	Median sternotomy (25)/Right thoracotomy (1)- On pump- Transatrial access	Felt strip and anchoring sutures	Mild (1)	4 ± 2	SAVR (11)
Sivan et al. [37] (USA)	2018	Sapien 3	1	Right thoracotomy -On pump-Transatrial access	Pledgeted anchoring sutures	No	-	-
Lupon et al. [38] (France)	2018	Sapien 3	1	Median sternotomy- On pump- Transatrial access	None	No	4.5	CABG
Russell et al. [13] (USA)	2018	Sapien 3	8	Median sternotomy On pump Transatrial access	PTFE strip	Trace (6) Mild (1)	-	SAVR (1) TVr (2)
Jeganathan et al. [39] (United Kingdom)	2019	Sapien 3	2	Median sternotomy On pump Transatrial access	Pledgeted anchoring sutures	No	Case 1: 2.7 Case 2: 7	TVr (1)
Morita et al. [40] (Japan)	2020	Sapien 3	1	Median sternotomy- On pump- Transseptal access	Use of felt strip and anchoring sutures	Mild	-	TVr
Albacker et al. [41] (Saudi Arabia)	2020	Sapien 3	1	Median sternotomy On pump Transatrial access	None	No	4	-
Tiwana et al. [42] (USA)	2020	Sapien XT and Sapien 3	40	Transapical (5) Transseptal (35)	None		5.5 ± 2.1	-

## Data Availability

The data underlying this study cannot be shared publicly in accordance with national data safety guidelines, to protect the privacy of individuals that included in the study. The data will be shared on reasonable request to the corresponding author.
